# Assessing Spatial Accessibility to Maternity Units in Shenzhen, China

**DOI:** 10.1371/journal.pone.0070227

**Published:** 2013-07-19

**Authors:** Peige Song, Yajie Zhu, Xi Mao, Qi Li, Lin An

**Affiliations:** 1 Department of Child, Adolescent and Women’s Health, School of Public Health, Peking University Health Science Center, Beijing, China; 2 Institute of Remote Sensing and GIS, School of Earth and Space Science, Peking University, Beijing, China; 3 Institute of Cartography and GIS, Chinese Academy of Surveying and Mapping, Beijing, China; University of Florida, United States of America

## Abstract

**Background:**

With the rapid development of urbanization, pregnant population is growing rapidly in Shenzhen, and it has been a difficulty to serve more and more pregnant women and reduce spatial access disparities to maternity units (MUs). Understanding of the current status of accessibility to MUs is valuable for supporting the rational allocation of MUs in the future.

**Methods:**

Based on pregnant population data and MUs data, this study uses a two-step floating catchment area (2SFCA) method based on Geographic Information System (GIS) to analyze the current spatial accessibility to MUs, and then make a comparison between that to public MUs and private MUs.

**Results:**

Our analysis of the accessibility to all MUs within a distance of 20 km shows that the accessibilities of the areas alongside the traditional border management line are acceptable, meanwhile highlights some critical areas, such as the west part of Nanshan district and the vast east part of Longgang district. The comparison between spatial accessibility to public MUs and private MUs shows statistically significant difference.

**Discussion:**

Results of this study suggest a great effort should be made to improve the equity of spatial accessibility to MUs in Shenzhen. For policy-making, strategy for the siting and allocation of future MUs, no matter public or private, should guarantee the greatest spatial accessibility for every pregnant woman.

## Background

**Figure 1:**
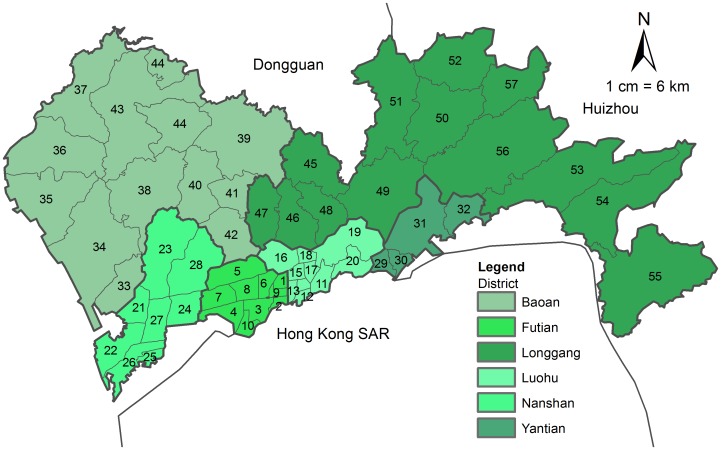
Administrative map of Shenzhen, China.

Shenzhen is a major city in Guangdong Province of China, situated immediately north of Hong Kong (Figure 1). In 1980, Shenzhen established the first Special Economic Zone (SEZ) in China and became the first experimental city of China’s reform and “opening up”. As a pioneer of developing socialist market economy, Shenzhen SEZ could use flexible governmental measures to develop economy and get strong economic policy support from central government of China. Over the past three decades, a lot of Chinese and foreign nationals have invested enormous amounts of money to develop manufacturing and service industries in Shenzhen SEZ, such as private real estates, private hospitals, etc. In the process of this experiment, the lessons from SEZ’s experience could be used in the rest of Chinese Mainland [1], [2]. With the rapid development of urbanization, Shenzhen became the first city without rural areas in China in 2010, which makes this study to be the first typical research of spatial accessibility to health resources in pure urban ground of China. From 2000 to 2010, Shenzhen’s urban population density increased from 3,596/km^2^ (in 2000, total population 7,008,428) to 8,588/km^2^ (in 2010, total population 10,357,938) [3], [4], and Shenzhen became the most crowded area in China and fifth in the World [5]. Its health system is already over-burdened, where hospital beds number (including long-term hospital beds, maternity beds and paediatric beds, but not delivery beds) per 1,000 population (a classic indicator to indicate the availability of inpatient services in terms of resources per population and reflect the status of an area’s health system) [6], [7] has decreased from 2.38 (in 2000) to 2.20 (in 2010) [3], [4], falling below the national average (latest in 2009, 4.20 beds per 1,000 population) [7]. Under this circumstance, it is important to know the current situation of health resources allocation in Shenzhen, and make reference for future urban construction and health resource planning.

As populations grow, the annual number of newborns per square kilometer (approximately 94/km^2^) in Shenzhen also continually ranks first in China recent years [8]. “Women and children’s health is the precondition and foundation of sustained human development” [9]. To ensure safe motherhood, every pregnant woman should accept professional antenatal examinations and skilled attendance at childbirth [10]. In obstetrics, longer travel distances may lead to accidental deliveries outside hospital [11] and may be associated with higher stillbirth rates [12]. In general, the WHO-recommended indicator-“Maternity beds density per 1,000 pregnant women” can be used to evaluate the gross access to maternity health care in an area [13]. Besides, a long waiting list or crowded waiting rooms may also reflect poor accessibility to delivery services, especially in urban areas [14]. According to Shenzhen Health and Population and Family Planning Commission, it is a great difficulty to find maternity beds in Shenzhen and pregnant women have to make an appointment of maternity beds in nearby maternity units (MUs) as soon as they are informed of pregnancy [15]. In order to make sure that the number of maternity beds in Shenzhen is sufficient and rationally located for all pregnant women and every woman can access skilled attendance when they deliver, the Shenzhen government is planning to encourage and guide the development of the private gynecological hospitals and make them to be an important complementary resource to public MUs. According to the latest statistic data from Shenzhen health statistics yearbook 2011 (2010 data), there are totally 81 hospitals with qualified MUs in Shenzhen, in which 52 (accounting for 64.2%) are public and 29 (accounting for 35.8%) are private [16]. In order to provide a more rational and efficient planning of MUs allocation, and guarantee the general equity of accessibility to MUs, policymakers need to have a good understanding of the current distribution pattern of supply and demand, and accessibility to MUs for pregnancy population.

Accessibility is a multidimensional concept, and researchers have defined it from various aspects. Bissonnette et al. emphasized the two components of access: potential and realized access. The former is the supply of health care resources, whereas the latter refers to the actual use of health services [17]. In addition, Peters et al.’s (2008) conceptual framework divided accessibility into four dimensions: availability of services, acceptability, financial accessibility and geographic accessibility [18]. These definitions above demonstrate multidimensionality of accessibility and emphasize the interaction of many factors in the process of access to health resources, including geographical, social, cultural, etc. This study focuses on physical/geographical accessibility, which can measure the spatial distribution of current health resources and assess equality of their distribution [17]. We aim to identify the current underserved areas and compare the different spatial accessibilities to public and private MUs.

There are many methods to measure spatial accessibility, such as regional availability [19], shortest path approach [20], the gravity-based method [14] and the two-step floating catchment area (2SFCA) method [21], [22]. The regional availability method is simply the ratio of health capacity to population within an area (generally referring to an administrative area), such as maternity beds per 1,000 pregnant population in one country (or province), and it is often used for comparison among different countries or regions [13]. It is a rough estimate of spatial access and cannot reveal the spatial variation within the target area. The shortest path approach calculates proximity between population and health service locations, not taking availability (supply) into account [20]. The gravity-based method, which concerns distance and demand relative to the supply, is a more realistic model than the shortest path approach because it takes both health service locations (supply) and population (demand) into consideration. It assumes that the attractiveness of a service diminishes with distance and associated increasing travel impedance [14], [20], and a continuous coefficient (β), which is also called travel friction coefficient, is used to represent distance impedance between demand and supply. One important limitation of this method is that it tends to conceal health professional shortage areas, which are precisely what we want to designate in study [21]. The 2SFCA method is a recently developed special case of gravity-based method which was first proposed by Radke and Mu [22], and then modified and improved by Luo and Wang [21]. It uses dichotomous distance impedance. First, it calculates the ratio of supply to demand within a service area centered at a supplier’s location and then sums up these ratios for demanders living in one area where different provider’s services overlap [23]. Through the computation, we can get an accessibility score for every predefined area (e.g., census tract), and this score can be straightforward to interpret because it essentially represents the total services shared by patients living in one area (a special form of beds-to-population ratio) [14], [21]. Comparing with the gravity-based method, the 2SFCA method was recommended by Luo and Wang because it is easier to interpret and has an advantage of identifying the poorly spatial access areas which are always the interest of planning and policy making to guarantee society welfare and equity of health resources [21].

This paper explores the distribution of MUs in Shenzhen by using the 2SFCA method, and constructs the appropriate research-scale for exploring the city MUs. Through the comparison of accessibilities to public and private MUs, we provide some advice on MUs planning in the urban area. The specific questions we attempt to answer are:

1. What is the current spatial distribution of MUs in Shenzhen?
2. Which regions are underserved and can be appropriate candidate areas for new MUs?
3. What’s the difference between the accessibilities to public and private MUs?

## Data and Methods

By the 2SFCA method, the accessibility of a particular census tract can be calculated depending on three main variables: the demand, the supply and travel distance.

### The Demand: Pregnant Population

In the first thirty development years (1980–2010) of Shenzhen, the border management line divided this city into two parts in terms of economic policy and development, the special economic zone (SEZ) part and the outside special economic zone (outside SEZ) part. The districts of SEZ were Nanshan, Futian, Luohu, Yantian and those outside SEZ were Baoan and Longgang. In 1 July 2010, the border management line was canceled, the SEZ was expanded to include all districts, and Shenzhen became an integrated SEZ from then on. Now, Shenzhen has six districts and each is further divided into sub-districts. As census tract in Shenzhen, sub-district is the lowest areal unit used for administrative management, and is thus chosen as the analysis unit in this study. There are totally 57 sub-districts in Shenzhen now (see Figure 1 and the corresponding name of each sub-district can be found in Table S1). In our study, the pregnant population refers to women who get pregnant and eventually give birth in Shenzhen MUs, and they are the actual users of the MUs. In Shenzhen, pregnant women are recommended to take antenatal examinations and give birth at a nearby MU by the government.

There is a WHO-recommended method for estimating the number of pregnant women from CBR (crude birth rate) [13]:

a = Estimated number of live births = (CBR per 1,000*total population);

b = Estimated number of pregnancies ending in stillbirths or miscarriages = (a * 0.15);

Estimated pregnancies expected in the year = (a+b);

In our study, we used an actual number of live births and stillbirths to represent pregnant population, which is much closer to the actual situation than the WHO-estimation method. We obtained the latest 2010 data of live births and stillbirths of each district to represent the amount of pregnant population from Shenzhen health statistics yearbook 2011 (2010 data) [16], and by assuming that the distribution of pregnant women is the same as that of total population, we got the pregnant population in each sub-district by multiplying the percentage of the sub-district population by each district population. In 2010, 95,726 pregnant women utilized maternity services in Shenzhen – about 0.9% of total population (10,357,938).

### The Supply: MUs

**Figure 2:**
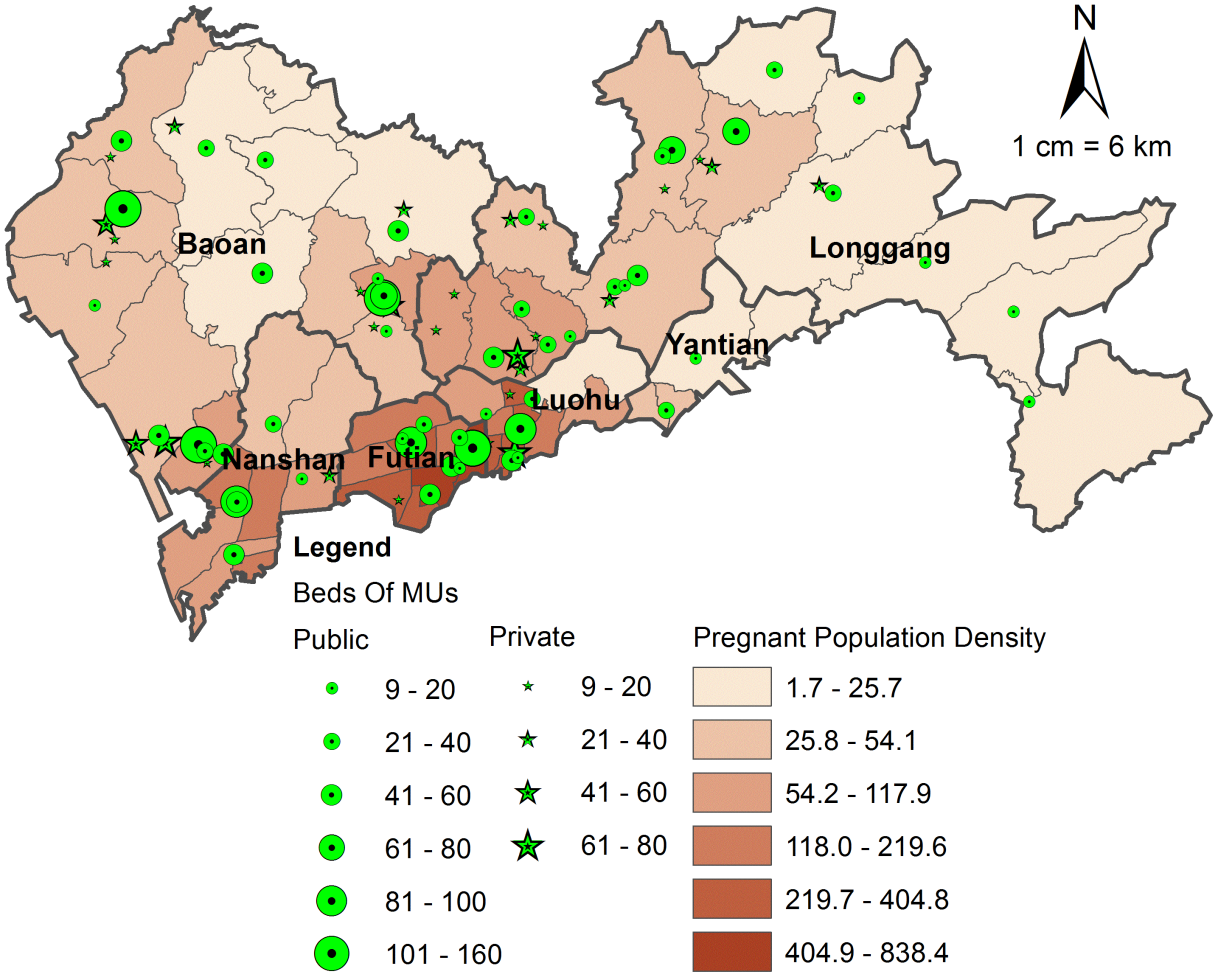
The spatial distribution of pregnant population and MUs in Shenzhen.

There are a total of 81 qualified MUs in Shenzhen, in which 52 (accounting for 64.2%) are public and 29 (accounting for 35.8%) are private (Figure 2). These MUs have maternity health services technology licenses, meeting the basic conditions of midwifery services in Shenzhen, and have passed the 2008 accreditation (once every three years) held by the administrative department in charge of health. These MUs can provide professional antenatal health examinations during pregnancy and skilled attendance at childbirth for pregnant women. There are many kinds of indicators to reflect a MU’s service capacity, such as maternity beds number, health staff number etc. [13], [24]. In this study, we chose maternity beds number to reflect the service capacity of a MU’s.

### Travel Distance

The travel distance between supply and demand is a key component to compute accessibility in the 2SFCA method. There are many indicators to represent travel distance, such as straight-line distance and travel time, etc. [25], [26], [27]. Travel time is a complicated indicator, which depends on road infrastructure, mode of transport and topography [28]. In this study, the detailed data of Shenzhen road network is unreachable for us, so we chose straight-line distance as a proxy for travel distance between the locations of supply and demand, and used it to delineate the search radius which represents the acceptable cut-off point. So far, there is no regulated or recommended straight-line distance threshold from a pregnant woman’s home to the locations of MUs, but there are two relevant time thresholds: a) according to the criteria for health professional shortage areas recommended by US Department of Health and Human Services, the specified threshold travel time for primary medical care should be 30 minutes in rural areas [29], [30]; b) according to a Netherlands research [31], the travel time from home to hospital should be within 20 minutes in order to avoid adverse outcomes in women at term. Considering that Shenzhen is an urban area and the regular antenatal health examinations during pregnancy are not emergency, travel time in those situations can largely exceed 20 minutes, so we set a time threshold as 30minutes In order to get the straight-line distance threshold which we actually need, we multiplied 30minutes and average road speed which was around 40 km/h in 2011 reported by Shenzhen Traffic Police Bureau, and get 20 km as the reference threshold straight-line distance value in this study.

### Methods

In this study, we used simple geographic centroids in each sub-district to represent pregnant population locations, and the information of MUs (including names, addresses and the number of maternity beds) was obtained from the website of Shenzhen Health and Population and Family Planning Commission [32]. We used the Baidu Map geocoding API (Version v1.0, Baidu Inc., Haidian, BJ) (http://developer.baidu.com/map/geocoding-api.htm) to obtain latitudes and longitudes of these MUs according to their street addresses, and then created a point layer (named “locations of MUs”) based on their coordinates using ArcGIS (Version 10.1, ESRI Inc., Redlands, CA). Afterwards, we transformed the coordinates from WGS-84 coordinate system to BJ-54 coordinate system.

The 2SFCA method was used in this study. There were two steps in calculating each census tract’s (sub-district’s) accessibility to MUs. First step, for each supply (MU) location j, search all demand (pregnant population) locations k that are within the threshold distance d_0_ (20 km) from location j, which forms the catchment area of supply (MU) location j, and then compute the maternity beds-to-pregnant population ratio R_j_ within this catchment area (equation 1):
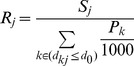
(1)where d_kj_ is the distance between locations k and j, S_j_ is the number of maternity beds of each MU (supply) at location j, and P_k_ is the amount of pregnant population (demand) whose location falls within the catchment area (d_kj_≤d_0_).

Second step, for each demand (pregnant population) location i, search all supply (MU) locations j that are within the threshold distance d_0_ (20 km) from location i, and then sum up all the maternity beds-to-pregnant population ratios R_j_ (derived in step 1) at those locations j to get the full accessibility A_i_^F^ at each demand location i (equation 2):
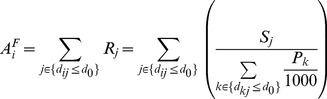
(2)where A_i_^F^ represents the full accessibility of each census tract to MUs, d_ij_ is the distance between i and j, R_j_ is the beds number of each MU to pregnant population ratio whose location j falls within the catchment area (d_ij_≤d_0_). The larger the value A_i_^F^, the better accessibility to MUs for one census tract’s pregnant population, which can also be interpreted that the more maternity beds are available for per 1,000 pregnant women under the threshold distance 20 km.

At the first step, the 2SFCA method computes the ratio of supply to demand within a threshold distance centered at supply’s location, at the second step, it sums up the initial ratios for demand in the overlapped service areas. With this method, we calculated each census tract’s accessibilities to all MUs, public MUs and private MUs respectively, and then compared the difference between accessibilities to public and private MUs using paired samples T-test. In order to set a basic level of accessibility and evaluate the conditions of these census tracts, we referred a WHO-recommended method [13]: under the assumption that there should be sufficient beds for all pregnant women within the threshold distance 20 km, the maternity beds occupancy rate was 80% (to account for the uneven spread of demand over time), and the mean duration of stay was 5.1 days (in 2010) [16], the target number should be (1000/.8) * (5.1/365) = 17.5 maternity beds per 1,000 pregnant women. Because accessibility score is a special pattern of beds per 1,000 population, we set 17.5 as the basic level of accessibility score. The sub-districts whose accessibility scores under 17.5 were the poorly served areas and those equal and above 17.5 were acceptable.

The spatial analysis with the 2SFCA method was carried out in ArcGIS (Version 10.1, ESRI Inc., Redlands, CA), and the statistical analysis of the accessibilities to public and private MUs was implemented by SPSS (Version 18.0, SPSS Inc., Chicago, IL).

## Results

Figure 2 depicts the spatial distribution of pregnant population at sub-district (census tract) level. The pregnant population density is relatively high in Nanshan, Futian, Luohu and the juncture area of four districts (Futian, Luohu, Baoan and Longgang). A declining tendency can be seen from the central part to the traditional outside SEZ. That is, it is more crowed in the central part of Shenzhen. The highest pregnant population density is about 838.4 pregnant women per km^2^ in a sub-district (Nanyuan) of Futian district. It is approximately 493 times higher than the least densely populated sub-district (Nanao) of Longgang district, whose pregnant population density is only about 1.7 women per km^2^. The huge variance is one of the most important factors which should be considered when making a reasonable allocation of new MUs.

Figure 2 also shows the spatial distribution of all MUs, including public MUs and private MUs. Consistent with the distribution of pregnant population, the MUs are centered on the most populated regions, such as Futian and Luohu. It also presents a declining tendency to the relatively vast east area. Except Longgang district, each of the other districts has more than one intermediate-to-large capacity (≥60 beds) MUs, most of which are public. Different from the public MUs, the private MUs generally are low-capacity, and tend to be centered in the juncture area of four districts (Futian, Luohu, Baoan and Longgang).

**Figure 3:**
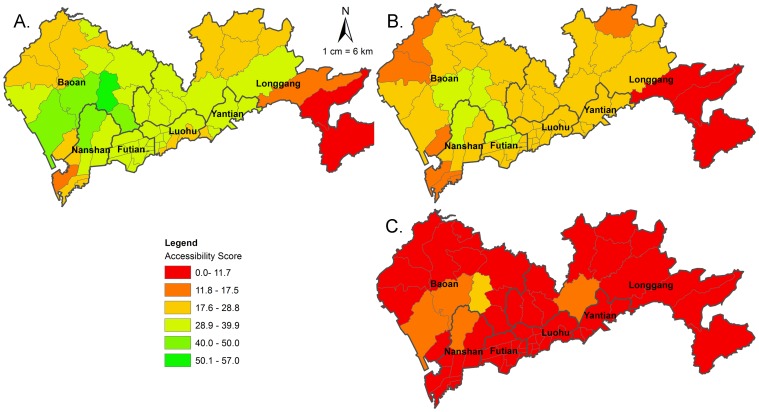
Accessibility to MUs in Shenzhen using the 2SFCA method. A. Accessibility to all MUs. B. Accessibility to public MUs. C. Accessibility to private MUs.

Figure 3 depicts the spatial accessibilities to all MUs, public MUs and private MUs. 1) The accessibility to all MUs shows significant variation, and the scores range from 6.5 to 57.0 with a mean value of 30.93 and a standard deviation of 8.54, which means that within a threshold distance of 20 km, there are averagely 30.9 maternity beds accessible for 1,000 pregnant women. The most accessible areas are located alongside a section of Baoan district’s edge which borders Nanshan and Futian. The others are centered on both sides of the traditional border management line and tend to decrease in the city boundary, especially in relatively vast but the least densely populated east sub-districts (Nanao, Dapeng and Kuiyong) of Longgang district, and west sub-districts (Nanshan and Shekou) of Nanshan district. 2) The accessibility scores to public MUs range from 6.5 to 39.4 with a mean value of 22.84 and a standard deviation of 6.16. 3) The accessibility scores to private MUs range from 1.8 to 17.6 with a mean value of 8.09 and a standard deviation of 2.91, and Nanao sub-district of Longgang district has no accessibility to private MUs (score = 0) within the distance of 20 km.

MUs consists of Public and private MUs. They are additional resources. For each sub-district, the sum of its accessibility scores to public and private MUs just equals to the score to all MUs. Through paired t test (Table 1), the difference between accessibility scores to public and private MUs is statistically significant (P<0.01).

**Table 1:** Comparison of accessibilities to public and private MUs.

	Accessibility		
	To Public MUs	To Private MUs	P
Sub-district (n = 57)	22.84±6.16	8.09±2.91	<0.001*

Abbreviation: MU, maternity unit. Data are shown as mean±standard deviation.* indicates a statistically significant difference.

## Discussion

In our study, the distribution of MUs was found to be consistent with the distribution of pregnant population, and clustered in the central part of Shenzhen, especially Futian and Luohu. Though the traditional border management line was cancelled in 2010, border effect still exists. People prefer living in the traditional SEZ, where market-oriented economy has been developed for a longer time, and therefore, economy, traffic, education, etc. are better. However, with the inburst of more and more migrant population these years, the migrant population size has already exceeded the resident population size. Because the house-renting and house-purchasing prices in traditional SEZ are higher than traditional outside-SEZ, many migrant people choose to live in the traditional outside-SEZ fringe zone alongside the traditional border management line [33], [34]. This concords with some previous studies [35], [36]. These factors may also affect the distribution of the self-financing private MUs, which are also centered in the juncture area of four districts (Futian, Luohu, Baoan and Longgang).

This study highlights critical areas in Shenzhen where new MUs or maternity beds may need to be added. This study shows that the accessibility scores (to all MUs) of sub-districts alongside the traditional border management line is acceptable, while the west part of Nanshan and the vast east part of Longgang have low accessibility scores (<17.5). These areas should be the areas of concern for further knowledge-based policy making. The comparison between accessibility scores to public MUs and to private MUs indicates that private MUs are important additional resources for public MUs. Attributing to the existing of private MUs, the mean accessibility score has increased from 22.84 (only public) to 30.93 (all). It also improves some census tracts’ accessibility scores and turns them to acceptable areas, such as the north parts of Baoan and Longgang. Whilst, the results also show that the private MUs are just additional but not complementary resources to public MUs, they own similar distributions. Thus, the function of private MUs is just strengthening the function of public MUs, not complementing the public MUs shortage areas.

Several limitations of this study should be addressed here. 1) The sub-district level pregnant population data is computed based on the percentage of the sub-district population by each district population. It is not accurate and real data to reflect the real world. 2) We used a single point as a proxy to represent the locations of pregnant population within each sub-district, and then calculated the distance from each pregnant population point to each MU, which may result in some aggregation-error [37]. 3) In this study, the bed number of each MU is obtained in 2012 from the Shenzhen Health and Population and Family Planning Commission website, it’s not the same year as the population data being used (2010). This may result in a little bias for the real accessibility conditions in 2010. 4) We set a fixed travel distance 20 km as the threshold distance. It means that all travel distance within 20 km are deemed equally accessible, while all travel distance beyond 20 km are not accessible. The choice of 20 km is applied to both steps 1 and 2, meaning that both the service catchment (breadth of population that a service is providing access to) and population catchment (breadth of services that a population has access to) are 20 km. The set of a signal buffer may result in under and overestimation of accessibility across census tracts, especially in the vast size sub-districts [20], [27]. Besides, in reality, though most pregnant women give birth in a nearby MU, sometimes, considering the seriousness of the condition, pregnant women or their families may accept a farther distance. As in other studies on spatial accessibility [23], [38], this study only took potential spatial accessibility into account, not revealed accessibility (or actual utilization of MUs). Thus, in the process of decision making of MUs allocation, the results need to be interpreted with caution. The accessibility to MUs is complicated, and many other aspatial aspects should be considered in addition to spatialityin order to reflect the revealed access or actual utilization of MUs, such as reputation, policy, etc.

Of course, our study has many advantages. 1) These years, the spatial accessibility methodology has become a popular tool in public health and city planning areas [39], [40], but in China, this study is the first to use a spatial method to assess accessibility to MUs. As an application of spatial methodology, this study illustrated the importance of incorporating the locations of MUs into the equitable and efficient allocation of health resources and city planning by providing a visualized and easy-understanding evidence for future planning of MUs. The result of this study will be useful for both health resource planners and other researchers in the field of public health. 2) The 2SFCA method based on GIS instead of the traditional rough indicators, such as service to population ratio or simple shortest distances [38], was chosen to determine areas that are outside of the pre-set catchment, and spatial accessibility was calculated at a much finer spatial resolution, thus the measurement of spatial accessibility was significantly advanced [23]. This is important and with big practical application value for Shenzhen, because taking antenatal examinations and giving birth at a nearby MU is strongly recommended by government [15]. Besides, the capabilities of geographic information systems (GIS) to handle large amounts of data over large geographic areas at fine levels of geographic detail make them suitable to measure geographical accessibility to medical clinics and other healthcare services [41], [42]. This study identified three east sub-districts (Nanao, Dapeng and Kuiyong) of Longgang district, and one west sub-district (Nanshan) of Nanshan district as the underserved regions, where health administrators and municipal planners should consider to be the potential areas for further MUs. 3) Through the comparison between accessibility scores to public MUs and private MUs, the evidence of how private MUs benefit the pregnant women of Shenzhen can be found. As the first city establishing socialist market economic system in China, Shenzhen has the most open and active private medical system, the market demand is the most important vane for private medical service planning. However, in this study, the complementary function of private MUs can’t be confirmed, their spatial distribution is similar to public MUs, and there are still some potential areas in great needs of maternity services. In recent years, the demand for maternity medical services is growing rapidly [43], and Shenzhen government encourages the development of private MUs. But how to allocate future public MUs and give reasonable guidance for the siting of future private MUs, this study is the first attempt in solving this problem, and our results will provide some advice on planning for both public MUs and private MUs. Furthermore, the research design and methods (including spatial access method, pregnant population calculation, the set of threshold distance and target accessibility score, etc.) in our study can also be applied to the other cities in China in the future.

## Supporting Information

Table S1 Accessibility to MUs in Shenzhen.
(DOCX)
