# Supplementary material for: Assessing Spatial Accessibility to Maternity Units in Shenzhen, China
Source: PLoS One. 2013 Jul 19;8(7):e70227. doi: 10.1371/journal.pone.0070227 (PMC3716609; doi:10.1371/journal.pone.0070227)
Supplement: Table S1 — Accessibility to MUs in Shenzhen. (DOCX) [file pone.0070227.s001.docx]

**Table S1.** Accessibility to MUs in Shenzhen.

|  |  |  | Accessibility |  |  |
| --- | --- | --- | --- | --- | --- |
| District | **Sub-district** | **ID** | **To All MUs** | **To Public MUs** | **To Private MUs** |
| Futian | Yuanling | 1 | 32.9 | 24.9 | 7.9 |
|  | Nanyuan | 2 | 33.4 | 26.0 | 7.4 |
|  | Futian | 3 | 32.4 | 26.0 | 6.4 |
|  | Shatou | 4 | 36.7 | 27.9 | 8.8 |
|  | Meilin | 5 | 39.3 | 29.6 | 9.7 |
|  | Huafu | 6 | 38.2 | 30.1 | 8.1 |
|  | Xiangmihu | 7 | 37.1 | 27.9 | 9.3 |
|  | Lianhua | 8 | 39.3 | 29.6 | 9.7 |
|  | Huaqiangbei | 9 | 33.9 | 26.0 | 7.4 |
|  | Fubao | 10 | 33.2 | 25.7 | 0.0 |
| Luohu | Huangbei | 11 | 27.9 | 20.5 | 7.4 |
|  | Nanhu | 12 | 28.5 | 21.1 | 7.4 |
|  | Guiyuan | 13 | 31.9 | 24.0 | 7.9 |
|  | Dongmen | 14 | 29.0 | 21.1 | 7.9 |
|  | Sungang | 15 | 29.0 | 21.1 | 7.9 |
|  | Qingshuihe | 16 | 30.1 | 22.2 | 7.9 |
|  | Cuizhu | 17 | 29.0 | 21.1 | 7.9 |
|  | Dongxiao | 18 | 29.4 | 21.1 | 8.3 |
|  | Donghu | 19 | 34.3 | 24.4 | 9.9 |
|  | Liantang | 20 | 27.3 | 20.2 | 7.1 |
| Nanshan | Nantou | 21 | 28.2 | 22.2 | 6.0 |
|  | Nanshan | 22 | 16.7 | 12.9 | 3.8 |
|  | Xili | 23 | 47.9 | 34.5 | 13.5 |
|  | Shahe | 24 | 34.2 | 25.6 | 8.6 |
|  | Shekou | 25 | 21.1 | 17.0 | 4.1 |
|  | Zhaoshang | 26 | 17.5 | 13.8 | 3.8 |
|  | Yuehai | 27 | 29.9 | 23.4 | 6.5 |
|  | Taoyuan | 28 | 38.4 | 28.3 | 10.1 |
| Yantian | Shatoujiao | 29 | 28.4 | 19.6 | 8.6 |
|  | Haishan | 30 | 31.3 | 20.8 | 8.8 |
|  | Yantian | 31 | 34.3 | 23.9 | 10.4 |
|  | Meisha | 32 | 31.0 | 24.9 | 10.6 |
| Baoan | Xinan | 33 | 25.9 | 17.3 | 9.7 |
|  | Xixiang | 34 | 40.4 | 28.6 | 11.8 |
|  | Fuyong | 35 | 32.2 | 21.9 | 13.0 |
|  | Shajing | 36 | 24.5 | 14.5 | 10.3 |
|  | Songgang | 37 | 20.2 | 13.1 | 10.1 |
|  | Shiyan | 38 | 43.3 | 30.3 | 10.5 |
|  | Guanlan | 39 | 32.0 | 23.9 | 8.7 |
|  | Dalang | 40 | 57.0 | 39.4 | 8.1 |
|  | Longhua | 41 | 35.6 | 27.5 | 7.1 |
|  | Minzhi | 42 | 41.2 | 31.4 | 8.1 |
|  | Gongming | 43 | 26.7 | 18.0 | 17.6 |
|  | Guangming | 44 | 29.7 | 19.2 | 7.9 |
| Longgang | Pinghu | 45 | 38.3 | 27.8 | 10.5 |
|  | Buji | 46 | 34.7 | 26.4 | 8.3 |
|  | Bantian | 47 | 32.5 | 24.3 | 8.3 |
|  | Nanwan | 48 | 39.3 | 28.7 | 10.7 |
|  | Henggang | 49 | 38.8 | 27.0 | 6.0 |
|  | Longgang | 50 | 26.6 | 19.9 | 11.8 |
|  | Longcheng | 51 | 27.3 | 18.8 | 8.4 |
|  | Pingdi | 52 | 22.2 | 17.4 | 4.9 |
|  | Kuiyong | 53 | 12.7 | 11.0 | 4.9 |
|  | Dapeng | 54 | 11.0 | 9.2 | 1.8 |
|  | Nanao | 55 | 6.5 | 6.5 | 1.8 |
|  | Pingshan | 56 | 29.6 | 24.1 | 6.7 |
|  | Kengzi | 57 | 23.5 | 18.6 | 5.5 |

Abbreviation: MU, maternity unit.
